# Diagnostic endoscopic ultrasound-guided fine-needle aspiration for disseminated parasitic leiomyomatosis after hysterectomy

**DOI:** 10.1055/a-2780-5973

**Published:** 2026-02-13

**Authors:** Koichi Soga, Ou Takagi, Haruka Kato, Masaru Kuwada, Ryosaku Shirahashi, Ikuhiro Kobori, Masaya Tamano

**Affiliations:** 1 26263 Department of Gastroenterology, Dokkyo Medical University Saitama Medical Center, Koshigaya, Japan

Parasitic leiomyomatosis, separate from the uterus with blood supplied via adjacent organs, is a rare extrauterine smooth muscle proliferation that arises spontaneously or after gynecological surgery 1 2 .

**Fig. 1:**
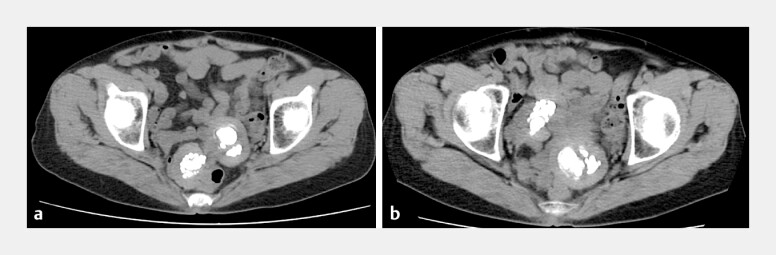
Unenhanced computed tomography (CT) images of the pelvis show calcified and mobile masses. **a** An unenhanced CT image of the pelvis 6 months prior to presentation to our hospital. **b** An unenhanced CT image of the pelvis immediately prior to referral to our hospital. Two lobulated pelvic masses with coarse calcifications were observed at the site corresponding to the previous surgical field 30 years after total abdominal hysterectomy and bilateral salpingo-oophorectomy for uterine myoma, endometriosis, and a ruptured chocolate cyst. The right-sided mass was adjacent to the intestinal tract, and a change in the position of the mass between **a** and **b** indicated the mobility of the lesion.

**Fig. 2:**
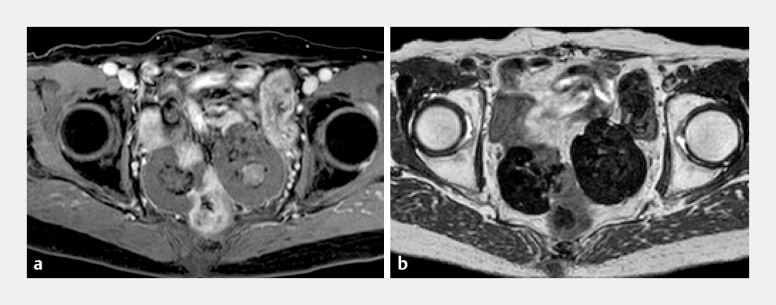
Magnetic resonance imaging (MRI) of the pelvis showing lobulated masses. **a** An axial contrast-enhanced T1-weighted MRI image (mDIXON water image). **b** An axial T2-weighted MRI image of the pelvis. **a** Fat-suppressed T1-weighted MRI images show areas with high signal intensity corresponding to the calcified foci observed with computed tomography (CT). **b** A T2-weighted MRI image of two lobulated pelvic masses with low signal intensity. The right-sided lesion is adjacent to the intestinal tract. A comparison with previous CT images revealed a positional change, suggesting a mobile lesion. These findings are most consistent with disseminated leiomyomatosis after hysterectomy.

A woman in her late 70s was referred to our gynecology department for further evaluation of two pelvic masses. She had undergone a total abdominal hysterectomy and bilateral salpingo-oophorectomy 30 years previously for uterine fibroids, endometriosis, and a ruptured ovarian endometriotic cyst. Abdominal computed tomography revealed coarse calcification within the mass lesions. However, although the right-sided mass appeared adherent to the bowel, a comparison using previously obtained images indicated a slight mobility and a non-infiltrative pedunculated nature ( Fig. 1 ). T2-weighted magnetic resonance imaging (MRI) of both lesions revealed low signal intensity and fat-suppressed T1-weighted MRI revealed a high signal intensity consistent with calcified or hyalinized components ( Fig. 2 ).

**Fig. 3:**
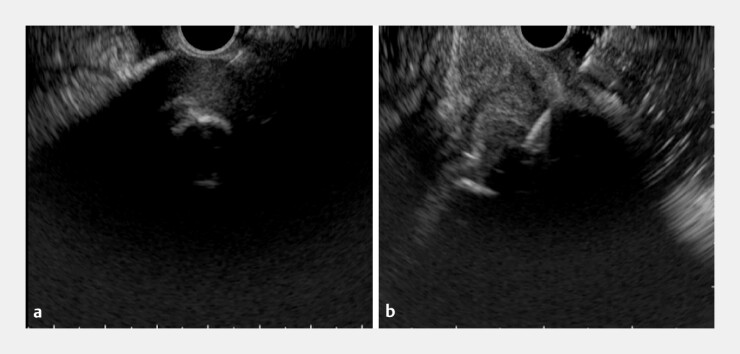
Endoscopic ultrasound (EUS) and EUS-guided fine-needle aspiration (EUS-FNA) findings for the pelvic mass. **a** An EUS image showing a well-circumscribed, homogeneous, hypoechoic mass with central calcification located adjacent to the rectal wall. The lesion was not adherent to surrounding structures and showed partial mobility during probe manipulation. **b** EUS-FNA was performed via the rectal route using a 19-gauge Trident needle under real-time guidance. The bladder was referenced as an anatomical landmark. The external layer of the lesion appeared uniformly hypoechoic, suggesting the fibrous tissue. Owing to the dense consistency and mobility of the tumor, aspiration using the slow-pull and suction techniques revealed the little firm material. A histopathological examination of the FNA specimen revealed hyalinized stromal tissue with an interlacing or bundled architecture without viable smooth muscle cells. Cellular atypia and mitotic figures were not observed. These findings are indicative of a hyalinized degenerative lesion, which was considered most likely to be a degenerated leiomyoma, without evidence of malignancy.

Endoscopic ultrasound (EUS)-guided fine-needle aspiration (FNA) was performed via the rectal route. EUS revealed a well-defined homogeneous hypoechoic mass with central calcification. The lesions were found to be characterized by partial mobility during probe manipulation, thereby indicating that they were not fixed to adjacent structures. For FNA, a 19-gauge needle was used to puncture the tumor under real-time guidance. The external layer of the lesion appeared hypoechoic and uniform, indicating the fibrous nature of the tissue. Given the dense consistency and mobility of the tumor, aspiration using the slow-pull and suction techniques yielded little firm material ( Fig. 3 , Video 1 ).

Diagnostic endoscopic ultrasound-guided fine-needle aspiration for disseminated parasitic leiomyomatosis after hysterectomy.Video 1

A histopathological examination of the FNA specimen revealed bundled and intersecting fascicles of smooth muscle cells with a hyalinized stroma. However, we detected no evidence of cellular atypia or mitotic figures. The final diagnosis of parasitic leiomyomatosis was based on the use EUS-FNA, which is rarely used for pelvic leiomyomatosis. EUS facilitated high-resolution imaging of the pelvic cavity and safe access to the mobile lesion under real-time guidance.

Endoscopy_UCTN_Code_CCL_1AD_2AJ
